# Jejuno-jejunal intussusception in a post-lung transplant patient from a gastrojejunostomy tube: A case report

**DOI:** 10.1016/j.ijscr.2019.01.034

**Published:** 2019-01-31

**Authors:** Takashi Harano, Pablo G. Sanchez, Graciela Bauza, John F. McDyer, Jonathan D’Cunha

**Affiliations:** a Department of Cardiothoracic Surgery, University of Pittsburgh Medical Center, Pittsburgh, PA, United States; b Department of Surgery, University of Pittsburgh Medical Center, Pittsburgh, PA, United States; c Division of Pulmonary, Allergy and Critical Care Medicine, University of Pittsburgh Medical Center, Pittsburgh, PA, United States

**Keywords:** GJ, Gastro-jejunostomy, Jejuno-jejunal intussusception, Gastro-jejunostomy tube, Surgical reduction of intussusception

## Abstract

Introduction: Gastro-jejunostomy tube is used for post-pyloric feeding for critical-ill patient who cannot tolerate oral alimentation. Jejuno-jejunal intussusception is a rare complication of gastrojejunostomy tube.

Presentation of case: A 39-year-old male with history of severe combined immunodeficiency, Achalasia and end-stage lung disease underwent double lung transplantation. After lung transplantation, he required gastrojejunostomy(GJ) tube placement due to his esophageal disease. Four days after gastrojejunostomy tube placement, he developed jejuno-jejunal intussusception. A 15 cm segment of thickened and enlarged bowel, which consisted of the intussusception were identified laparoscopically. Surgical reduction was performed without bowel resection.

Discussion: Intussusception is uncommon in adults compared to pediatric population. In this rare case, the jejunal limb of the GJ tube placed in jejunum was the cause of jejunojejunal intussusception serving as the lead point. The GJ tube should not be placed farther down from ligaments of Treiz to prevent jejuno-jejunal intussusception.

Conclusions: A heightened index of suspicion for this rare complication should exist with a presenting patient has signs of proximal bowel obstruction and CT evidence of intussusception.

## Highlights

• Jejuno-jejunal intussusception is a rare complication of gastrojejunostomy tube.
• Surgical reduction should be performed when intussusception is resistant to conservative management.
• When the gastrojejunostomy tube is placed in free jejunum, the tube can be a lead point for intussusception. The gastrojejunostomy tube should not be placed farther down from ligaments of Treiz to prevent jejuno-jejunal intussusception.

## 1 Introduction

Enteral feeding is essential for the care of critical-ill patient. Both gastric and jejunal tube feeding are effective methods for providing nutrition to patients who cannot tolerate oral alimentation. However, post-pyloric tube feeding can lower the risk of pneumonia than gastric tube feeding [1]. Our clinical pathway after lung transplantation for patients with severe gastroesophageal reflux disease and esophageal dysmotility includes being NPO for 2 months and Gastrojejunostomy (GJ) tube with jejunal feedings [2].

Intussusception is uncommon in adults compared to pediatric population. In general, half of the adult intussusception are associated with tumors in intestine [3]. In those case the tumors serve as the lead point of intussusception. There are several reports commented about jejunal intussusception related to long intestinal tubes [[3], [4], [5]]. We have experienced a case of jejuno-jejunal intussusception in which the jejunal limb of GJ tube served as this lead point.

The work has been reported in line with the SCARE criteria [6].

## 2 Presentation of case

**Fig. 1:**
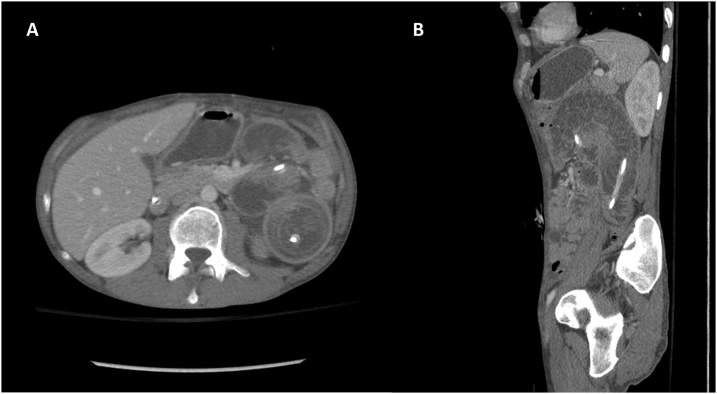
(A) Abdominal CT showed Jejunojejunal intussusception with distension of stomach and duodenum. (B) Gastrojejunal tube was passed through intussusception.

**Fig. 2:**
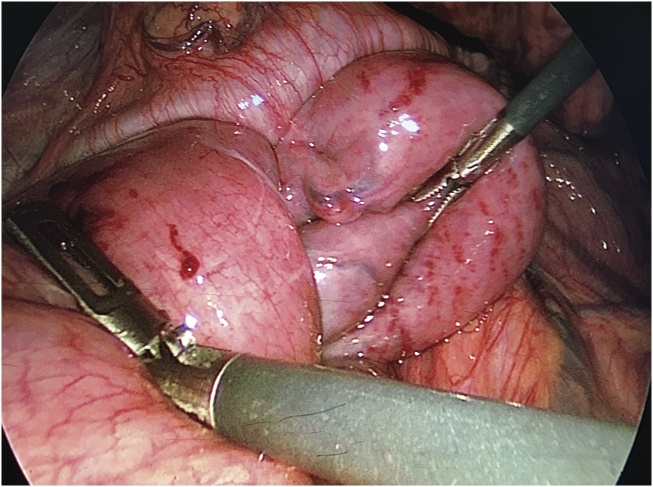
The intussuscepted jejunum was started about 5 cm from the ligament of Treitz into the distal jejunum. We were able to pull out the proximal jejunum from the distal jejunum.

A 39-year-old male with history of severe combined immunodeficiency who had underwent bone marrow transplant in early childhood, underwent bilateral sequential lung transplantation for end-stage lung disease due to recurrent pneumonia and graft-versus-host-disease. He also had a long-standing history of achalasia. He had a history of Heller myotomy as a small child and his esophagus was previously dilated 4 times prior to lung transplantation due to dysphagia. He was malnourished and BMI was 16.3 when he was listed for lung transplantation. He underwent bilateral sequential lung transplantation on cardiopulmonary bypass. Immediate post-lung transplant course was uneventful. He received his tube feeding from post pyloric feeding tube and he underwent percutaneous gastrostomy tube placement 2 weeks after lung transplantation. A jejunal tube was wired through the gastrostomy tube to provide a functional GJ tube to reduce aspiration risk. The tip of jejunostomy tube was placed at 30 cm distal from ligament of Treiz. This is a standard component to our post operative clinical pathway for patients with esophageal and reflux issues. He tolerated his feedings well and was discharged. Four day after replacement of GJ, he returned to emergency department complaining nausea, vomiting, and vague abdominal pain. Abdominal CT showed jejuno-jejunal intussusception with distension of stomach and duodenum. GJ tube was noted to pass through the area of intussusception thereby acting as the lead point which is classic for the pathology of intussusception (Fig. 1). His jejunal tube was removed and the gastrostomy tube placed to gravity drainage. His nausea was improved after exchanging tube. Follow-up abdominal CT was performed 24 h after removal of gastrojejunostomy tube which showed persistent jejuno-jejunal intussusception and he remained mildly symptomatic with pain. He was taken to the operating room for surgical reduction of jejuno-jejunal intussusception. Laparoscopically we could identify a 10–15 cm segment of thickened and enlarged bowel, which consisted of the intussusception. The intussuscepted jejunum started about 5 cm from the ligament of Treitz and proceeded distally. We could slowly pull out the proximal jejunum from the distal jejunum (Fig. 2). We ensured all the jejunum was viable and without injury. His post op course was uneventful. We converted his gastrostomy tube back to unibody GJ tube on postoperative day 5 and started tube feeding on the following day. The tip of the GJ tube was placed at the level of ligament of Treitz to prevent jejuno-jejunal intussusception. He is doing well in clinical follow-up and awaiting definitive management of his achalasia.

## 3 Discussion

Intussusception is uncommon in adults compared to pediatric population. In general half of the adult intussusception is associated with tumor in intestine [1]. There are several reports commented about jejunal intussusception related to long intestinal tube [[3], [4], [5]]. In this rare case, the jejunal limb of the GJ tube was the cause of jejunojejunal intussusception. The jejunum requires a lead point for intussuception and the jejunal limb of GJ tube placed in jejunum served as the lead point. Hughes et al. [5] reported jejunal intussusception related to GJ tube in pediatric patients. Initially they preferred to use pigtail catheter as a GJ tube. They suspected that pigtail catheter increased the risk for jejunal intussusception as distal pigtail part could play a role to increase the risk for intussusception serving as a lead point. When they applied short straight catheter instead of pigtail catheter for patients who had intussusception, the recurrence rate was lower. Others have also reported this complication in adults [3,5,7]. Carucci et al^7^] reported that focal intussusception of the jejunum was identified at the level of the jejunstomy tube in 4 of 280 (1%) cases and they also commented that the tube itself presumably served as the lead point for the intussusception. When the GJ tube placed in free jejunum away from ligaments of Treiz, the GJ tube could serve as a lead point with tucking the free jejunum around the tube with its motilities. The GJ tube should not be placed farther down from ligaments of Treiz to prevent jejuno-jejunal intussusception.

## 4 Conclusion

This report introduced the case of adult jejuno-jejunal intussusception associated with GJ tube following lung transplantation. A heightened index of suspicion for this rare complication should exist with a presenting patient has signs of proximal bowel obstruction and CT evidence of intussusception.

## Conflicts of interest

The authors declare that they have no competing interests.

## Funding

This research did not receive any specific grant(s) from funding agencies in the public, commercial, or not-for-profit sectors.

## Ethical approval

I certify that this kind of manuscript does not require ethical approval by the Ethical Committee of our institution.

## Consent

Written informed consent was obtained from the patient for publication of this case report. A copy of the written consent is available for review by the Editor-in-Chief of this journal on request.

## Author contribution

All authors were involved in the care of the patient. TH, GB, and JD performed the surgery. TH wrote the draft with supervision of JD. PS, JM and JD edited. All authors have read and approved the final manuscript.

## Registration of research studies

N/A.

## Guarantor

Takashi Harano.

## Provenance and peer review

Not commissioned, externally peer-reviewed.

## References

[1] Alkhawaja S., Martin C., Butler R.J., Gwadry-Sridhar F. (2015). Post-pyloric versus gastric tube feeding for preventing pneumonia and improving nutritional outcomes in critically ill adults. Cochrane Database Syst. Rev..

[2] Crespo M.M., Bermudez C.A., Dew M.A. (2016). Lung transplant in patients with scleroderma compared with pulmonary fibrosis. Short- and long-term outcomes. Ann. Am. Thorac. Soc..

[3] Wang N., Cui X.-Y., Liu Y. (2009). Adult intussusception: a retrospective review of 41 cases. World J. Gastroenterol..

[4] Gayer G., Zissin R., Apter S., Papa M., Hertz M. (2002). Pictorial review: adult intussusception—a CT diagnosis. Br. J. Radiol..

[5] Hughes U.M., Connolly B.L., Chait P.G., Muraca S. (2000). Further report of small-bowel intussusceptions related to gastrojejunostomy tubes. Pediatr. Radiol..

[6] Agha R.A., Fowler A.J., Saeta A. (2016). The SCARE statement: consensus-based surgical case report guidelines. Int. J. Surg. Lond. Engl..

[7] Carucci L.R., Levine M.S., Rubesin S.E., Laufer I., Assad S., Herlinger H. (2002). Evaluation of patients with jejunostomy tubes: imaging findings. Radiology.

